# Elevation-induced changes in soil sulfur availability in tea plantations

**DOI:** 10.3389/fpls.2025.1624346

**Published:** 2025-08-07

**Authors:** Hong Wang, Chen Qian, Hiba Shaghaleh, Jianfei Wang, Xiaoliang Li, Sumei Duan, Cece Qiao, Yousef Alhaj Hamoud

**Affiliations:** ^1^ Engineering Research Center for Smart Crop Planting and Processing Technology, Anhui Science and Technology University, Chuzhou, China; ^2^ School of Energy and Environment, Southeast University, Nanjing, China; ^3^ College of Hydrology and Water Resources, Hohai University, Nanjing, China

**Keywords:** tea plantation, cycling process, stoichiometry, available sulfur, soil nutrient

## Abstract

Chinese tea plantations, as the world’s leading tea producers, face escalating challenges such as soil acidification and nutrient management. Investigating soil nutrient variations along elevation gradients is crucial. Despite extensive research on macronutrients like carbon, nitrogen, and phosphorus, the role of available sulfur (AS), tightly interlinked with other nutrients, remains underexplored. This study focuses on available S dynamics in Huoshan County, Anhui Province, utilizing soil and litter samples collected from tea plantations at diverse elevations. The results revealed non-linear variations of soil AS with altitude and principal component (PC1) of other soil properties, significantly influencing tea plantation segregation by elevation. Available S exhibited heightened sensitivity to elevation changes compared to other nutrients, underscoring its pivotal role in tea plantation management and soil nutrient cycling. Furthermore, tea plantation dimensions notably decreased with increasing altitude. These findings emphasize the importance of available S in tea garden nutrient management and suggest its crucial consideration in future research and management endeavors. The non-linear correlation between available S and PC1 highlights the responsiveness of available S to elevation variations, emphasizing its significance in tea plantation soil dynamics. This study offers valuable insights into optimizing nutrient management strategies in tea plantations amidst elevation gradients.

## 1 Introduction

Tea (*Camellia sinensis* (L.) O. Kuntze), belonging to the genus Camellia in the Camelliaceae family, is a shade-loving, economically significant cash crop widely cultivated in developing countries, including China, India, and Sri Lanka. In 2019, global tea production reached 6.5 million tons, with over 5.08 million hectares under cultivation. Of which, China had a planted area of 3.18 million hectares, representing 62% of the global tea cultivation area, making it the world’s largest tea producer. The substantial economic value and regional characteristics of the tea industry make it a vital component of China’s industrial poverty alleviation and rural revitalization efforts. Achieving high tea yield is a key objective in tea garden management, and soil, serving as the nutrient reservoir for tea plants, plays a crucial role in their growth.

Soil acidification in tea gardens is an escalating global problem affecting tea production (Mupenzi et al., 2011). Soil pH is a critical factor influencing plant growth. Besides being affected by the source of soil parent material and acid deposition (Oh et al., 2006), artificial factors such as fertilizer application (Zakaria et al., 2025) and mass crop production (Ok et al., 2007) also affect soil pH, posing challenges to sustainable agricultural development. The optimal growth pH for tea plants ranges from 4.5 to 6.0, with 5.5 being ideal (Tang et al., 2019). When the pH drops below 4, tea plant growth is restricted, affecting yield and quality (Lin et al., 2022) and potentially endangering human health (Ju et al., 2024). Conversely, a pH above 6.5 can halt tea plant growth, with levels above 7 leading to plant death (Lin et al., 2022). Soil acidification not only results in nutrient loss (Alekseeva et al., 2010) but also compromises tea safety (Yang et al., 2025). In addition, soil pH is a vital characteristic in determining the chemical environment of higher plants and soil microbes. Tea cultivation uniquely contributes to soil acidification over time, with the pH decreasing as the duration of tea planting increases.

Soil acts as a major reservoir for carbon and nitrogen (Brooker et al., 2014; Yang et al., 2025). In tea plantations, soil nutrient input primarily derives from litter decomposition, with tea plants accumulating a significant amount of aluminum (Sun et al., 2020). Soil acidification in tea gardens is mainly due to the biological aluminum accumulation by tea trees, increased exchangeable aluminum and aluminum complexes, and soil base leaching (Hu et al., 2017). While extensive research has focused on macronutrients carbon (C), nitrogen (N), phosphorus (P), and potassium (K) in tea gardens (Pang et al., 2019), studies on sulfur (S) have been limited. Sulfur is essential for the growth and development of organisms, accounting for approximately 1% of an organism’s dry mass (Gebauer et al., 1994). Soil S availability and its cycling may change in response to variations in other major nutrients (Schmidt et al., 2011). The stoichiometric relationships between C, N, P, and S suggest that changes in one element can impact the cycling of others (Tipping et al., 2016). For example, nitrogen deposition can alter soil N use efficiency, affecting the S cycle and other related nutrients (Chen et al., 2016). S is also critical in protein formation, enzyme function, and nutrient interactions (Shi et al., 2016; Wilhelm Scherer, 2009).

Research on soil S has primarily focused on grassland (Nguyen and Goh, 1992), forest (Fan et al., 2017), and farmland (Wang et al., 2005), investigating aspects such as N deposition effects on the S cycle (Chen et al., 2016), the role of the S cycle in plant metabolism, and S deposition (Fan et al., 2017). In tea gardens soil eco-stoichiometry studies, S has often been overlooked. Given that China’s forests are limited by P and S, and these limitations may increase (Hu et al., 2017), it is imperative to research S in tea gardens.

Elevation is an important environmental factor influencing plant distribution, growth, and soil properties. Investigating the availability of S and the growth of tea plants at varying elevations is essential for understanding the adaptability of tea plants to altitude and their physiological mechanisms. Lower elevations generally experience higher temperatures and evapotranspiration, making plants susceptible to drought stress (Yang et al., 2025). As elevation increases, the environmental water status may improve, but higher elevations can lower soil metabolic rates and shorten growing seasons due to cooler temperatures, thus limiting the growth of plants (Körner, 2021; Vospernik and Nothdurft, 2018).

The distribution characteristics of S in tea gardens and its relationship with other soil elements still need to be explored, limiting our understanding of nutrient cycling in these ecosystems. Furthermore, significant human disturbance in tea plantations poses additional challenges. Given the importance of S in soil element cycling and plant growth, understanding the interactions between soil S and other elements and their responses to altitude will enhance our knowledge of nutrient distribution in tea gardens at different altitude gradients. This understanding provides a theoretical basis for effective nutrient management in tea plantations.

We hypothesize that soil available S content would vary significantly with elevation due to changes in the soil properties and nutrient characteristics, which in turn affect the bioavailability and cycling of S (Sundqvist et al., 2013). This study aims to answer the following questions: What are the distribution characteristics of available S in tea garden soils at different altitudes? What is the relationship between S and the basic physical and chemical properties and nutrient characteristics of the soil? This study not only analyzed the vertical differentiation mechanism of sulfur biogeochemical cycling in mountainous tea gardens, but also provided scientific targets for precision fertilization, ecological restoration, and quality control in tea gardens by quantifying the quantitative relationship between altitude and sulfur availability. At the same time, it deepened the theoretical understanding of material cycling in mountainous ecosystems under the background of global change.

## 2 Materials and methods

### 2.1 Study sites

This study was conducted on tea plantations in Huoshan County, Anhui Province, China (115°52′–116°32′E, 31°03′–31°33′N). The research area is characterized by yellow-brown soil and a north subtropical humid monsoon climate, providing abundant rainfall, light, water, and heat resources. The study sites are primarily located in low-altitude areas. The annual average temperature is approximately 15.3°C, and the annual average precipitation reaches up to 1366 mm.

### 2.2 Experimental design

The experiment commenced in June 2016. Soil samples from tea gardens were collected during seven days without rain. Sampling elevation ranges, and gradient divisions were determined based on the natural distribution of tea tree planting in the study area. The samples were taken from three elevation ranges: 100–500 m, 500–600 m, and above 600 m, with the maximum elevation not exceeding 700 m.

The elevation bands (100–500 m, 500–600 m, >600 m) were determined based on the topographic and ecological characteristics of the study area, ensuring each band represents a distinct environmental zone. Though the intervals are uneven, they were selected to reflect meaningful ecological transitions and minimize intra-band variability. Statistical adjustments were applied to account for area differences across bands.

### 2.3 Sample collection

**Figure 1:**
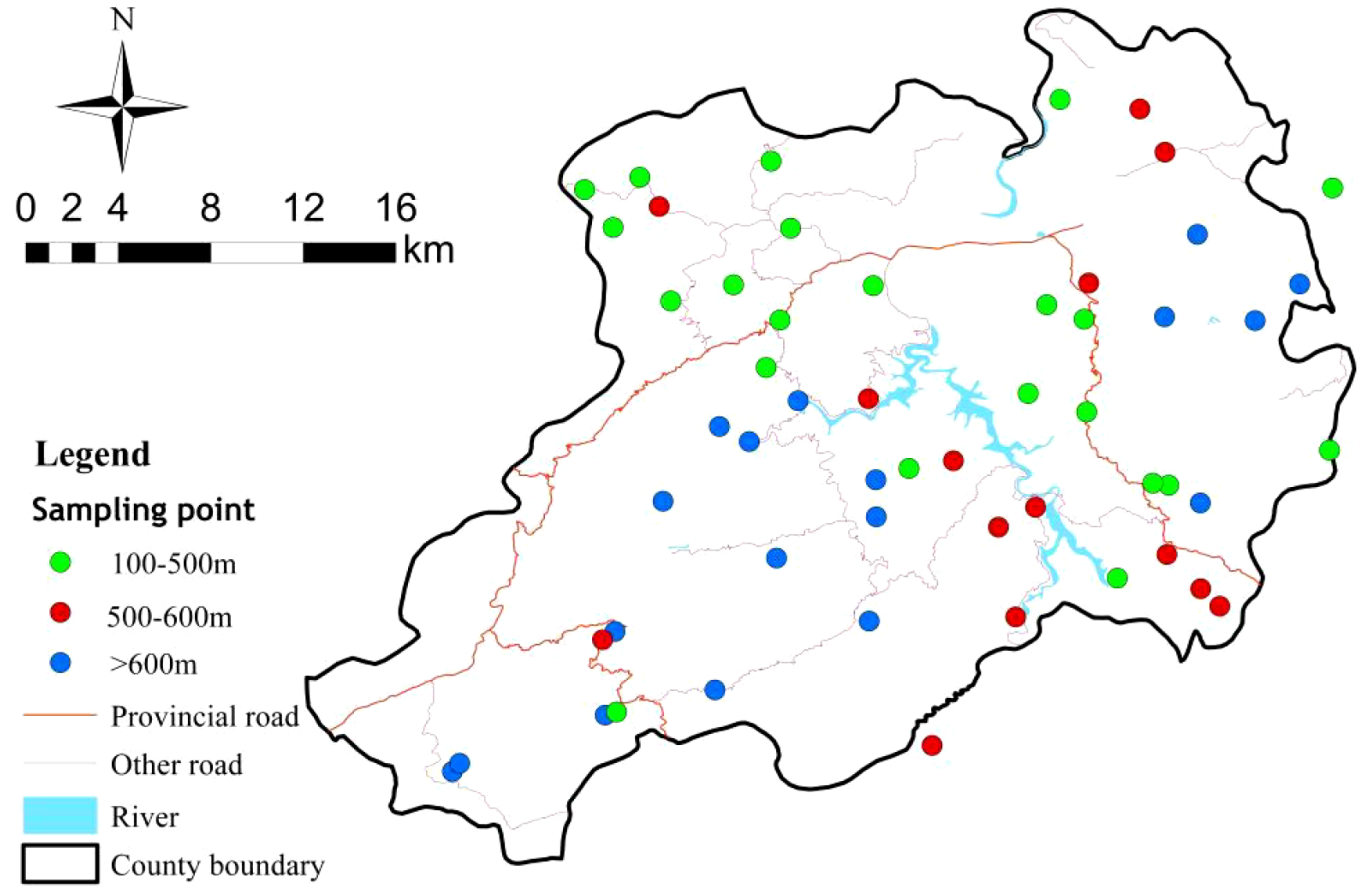
Geographic location of sampling sites.

A series of sampling points were selected within the study area (**Figure 1**). These points are distributed across three elevation ranges: 100–500 m, 500–600 m and > 600 m, and the sampling numbers were 22, 14, 18, respectively. The average horizontal distance between sampling points were 100meters. All plots were positioned on similar slopes and slope directions to eliminate heterogeneity caused by terrain variations. Soil samples were collected from five randomly selected points within each plot at a depth of 0–20 cm. The samples were mixed and transported to the laboratory in an aluminum boxes, where they were air-dried and stones and debris were removed. The air-dried soil samples were sieved through a 2 mm sieve for available nutrient analysis and Mg, a 0.149 mm sieve for elemental determination including S and Cu. The soil samples were dried to a constant weight to measure soil moisture. Simultaneously, litter from the tea plantations was collected at each elevation sampling point during the soil sampling. Care was taken to avoid already decomposed litter. The collected litter was brought back to the laboratory and powdered for chemical property analysis. This comprehensive collection approach ensures that both soil and litter samples provide a thorough understanding of the nutrient dynamics within the tea plantations at different elevations. 

### 2.4 Soil nutrient and plant growth measurement

Air-dried soil samples were passed through a 2 mm sieve for pH determination using a glass electrode; 1:2.5, soil/water ratio and available P analysis using a flow injection auto analyzer (Smartchem 200 Alliance Corp. France) (Xie et al., 2019). Samples passed through a 0.149 mm sieve were digested using a digester (FOSS. Tecator Digestor 20), and the total nitrogen content of soil (SN) and litter samples (TN) was measured with a flow injection auto analyzer. Soil organic C (SOC) and total organic C (TOC) of litter were determined using the potassium dichromic (H_2_SO_4_-K_2_Cr_2_O_7_) method. The total phosphorus (TP) of the litter was measured after digestion with H_2_SO_4_. Available magnesium (Mg) was extracted using a 2 mol L^−1^ KCl solution and determined by atomic absorption spectrometry. Available potassium (K) was extracted by a 0.2 mol L^−1^ NaHCO_3_ + 0.01 mol L^−1^ EDTA + 0.01 mol L^−1^ NH_4_F and measured using atomic absorption spectrometry. Available S was extracted with 0.01 mol L^−1^ Ca_3_(PO_4_)_2_ and determined using a turbidimetric procedure (Schmidt et al., 2011). Available copper (Cu) was extracted with 0.005 mol L^−1^ DTPA at pH 7.30 and determined by inductively coupled plasma atomic emission spectrometry (ICP-AES).

Tea plantation growth was assessed by measuring the height and width of the plants at different altitude gradients using a meter ruler. The ground diameter of the tea plants was measured using a diameter at breast height (DBH) ruler. Average values are calculated for each measurement.

### 2.5 Statistical analysis

A second-order polynomial function was used to examine the relationship between available S and pH, SOC, SN, available Mg, available P, and available K. This function was also used to determine the relationship between available S and the dominant soil nutrient gradient estimated by principal component analysis (PCA). SOC, SN, pH, and all measured soil available nutrients except available S were used for PCA to obtain the structure. The discriminant analysis examined whether plots from different elevation ranges could be separated based on SOC, SN, pH, and five available soil nutrients. Pairwise correlation analysis determined the variables contributing most to these separations. One-way analysis of variance (ANOVA) examined the effects of elevation on soil and litter chemical properties and on eight, crown width, and ground diameter. *Post hoc* Student’s t-tests were conducted for multiple comparisons. All statistical analyses were performed using JMP 9.0 (SAS Institute, Cary, NC, USA) and the significance was *P*<0.05.

## 3 Results

### 3.1 Changes in tea plant growth at different altitudes

The plant height and width of tea plantations showed a decreasing trend with increasing altitude (**Table 1**). Compared to plants height at 100–500 m and 500–600 m, those at > 600 m exhibited 5.59% and 5.12% reduction, respectively. The height of tea plants at 100–500 m was similar to that at 500–600 m. Regarding width, plants at > 600 m were 6.00% narrower compared to those at 100–500 m and 5.62% narrower compared to those at 500–600 m. Again, the difference in width between 100–500 m and 500–600 m was insignificant. The diameter of tea plants also decreased with the increasing altitude, but this decrease was not significant.

**Table 1:** Plant height (cm), plant width (cm), and diameters(cm) of tea plantations at different elevation ranges.

Elevation	Plant height (cm)	Plant width (cm)	Diameters (cm)
100–500 m	105.25 ± 2.06A	65.75 ± 1.25A	13.25 ± 0.95A
500–600 m	100.25 ± 1.80A	63.25 ± 1.11A	11.75 ± 0.63A
>600 m	100.75 ± 2.75A	62.50 ± 1.04A	11.00 ± 0.58A

Means ± standard error of varying elevation ranges are shown in the table. Means with the same letter indicate no significant difference in *post-hoc* tests.

### 3.2 Distribution of sulfur in tea plantations at different altitudes

**Figure 2:**
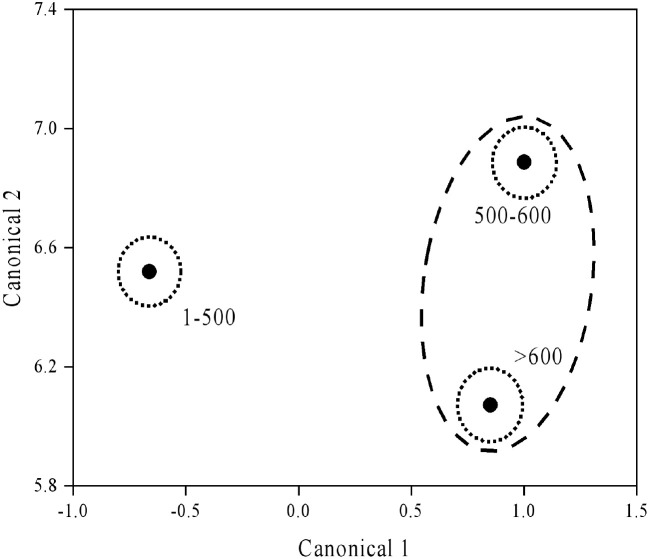
Discriminant analysis ordination of tea plantations with different elevations.

The content of available P, K, and S increased with elevation. On the contrary, changes in other major nutrients with elevation were not consistent, with soil pH ranging between 4.12–4.31. The highest content of SN and available Mg were observed at 100–500 m, while the lowest content was at 500–600 m. There was no significant difference in the TOC, TN, litter TP, SOC, or available Cu across different altitudes (**Table 2**). Canonical discriminant analysis suggested substantial separation of tea plantation by elevation gradient (**Figure 2**); follow-up analysis showed that soil pH, available Mg, and S contributed to Canonical 1, with available S being the most influential. Soil pH and available Mg significantly contributed to Canonical 2, with available Mg contributing the most.

**Table 2:** Soil pH, total C and content (g kg^−1^), available nutrients (mg kg^−1^), and plant total organic carbon (TOC, g kg^−1^), plant total nitrogen (TN, g kg^−1^), and plant total phosphorus (TP, g kg^−1^) of tea plantation at different elevation ranges.

Variable	Elevation (m a.s.l.)		
	100–500 m	500–600 m	>600 m
Soil			
pH	4.12 ± 0.03C	4.31 ± 0.11A	4.19 ± 0.07B
SOC (g kg^−1^)	29.33 ± 2.31A	28.46 ± 2.81A	28.89 ± 1.79A
SN (g kg^−1^)	1.00 ± 0.07A	0.95 ± 0.09C	0.97 ± 0.06B
Available P (mg kg^−1^)	34.75 ± 9.59B	43.01 ± 9.53A	47.74 ± 8.16A
Available K (mg kg^−1^)	72.46 ± 6.28B	80.46 ± 12.17AB	82.40 ± 5.87A
Available Mg (mg kg^−1^)	122.60 ± 20.58B	213.82 ± 36.81A	144.85 ± 13.97B
Available S (mg kg^−1^)	20.74 ± 1.56A	16.42 ± 1.22B	15.98 ± 0.57C
Available Cu (mg kg^−1^)	1.17 ± 0.20A	1.25 ± 0.16A	1.16 ± 0.13A
Litter			
TOC (g kg^−1^)	426.90 ± 6.01A	419.57 ± 6.27A	412.08 ± 5.75A
TN (g kg^−1^)	15.28 ± 0.60A	14.38 ± 0.46A	13.71 ± 0.33A
TP (g kg^−1^)	1.24 ± 0.04A	1.24 ± 0.05A	1.17 ± 0.08A

Means ± standard error of varying elevation ranges are shown in the table.

Means with the same letter indicate no significant difference in *post-hoc* tests.

### 3.3 Relationship between soil S and other elements in tea plantations

**Figure 3:**
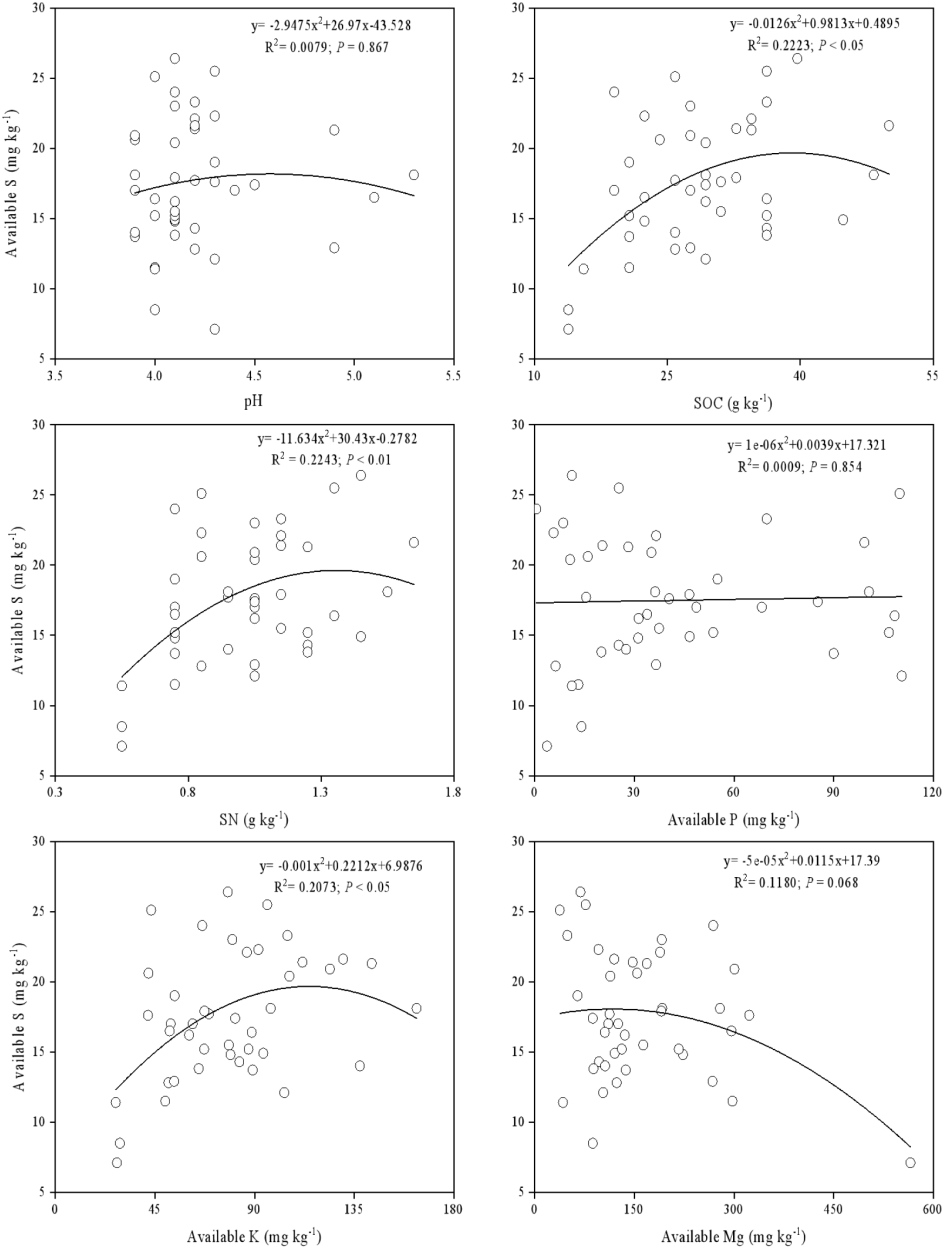
Correlations between available S and five major nutrients and soil pH.

**Figure 4:**
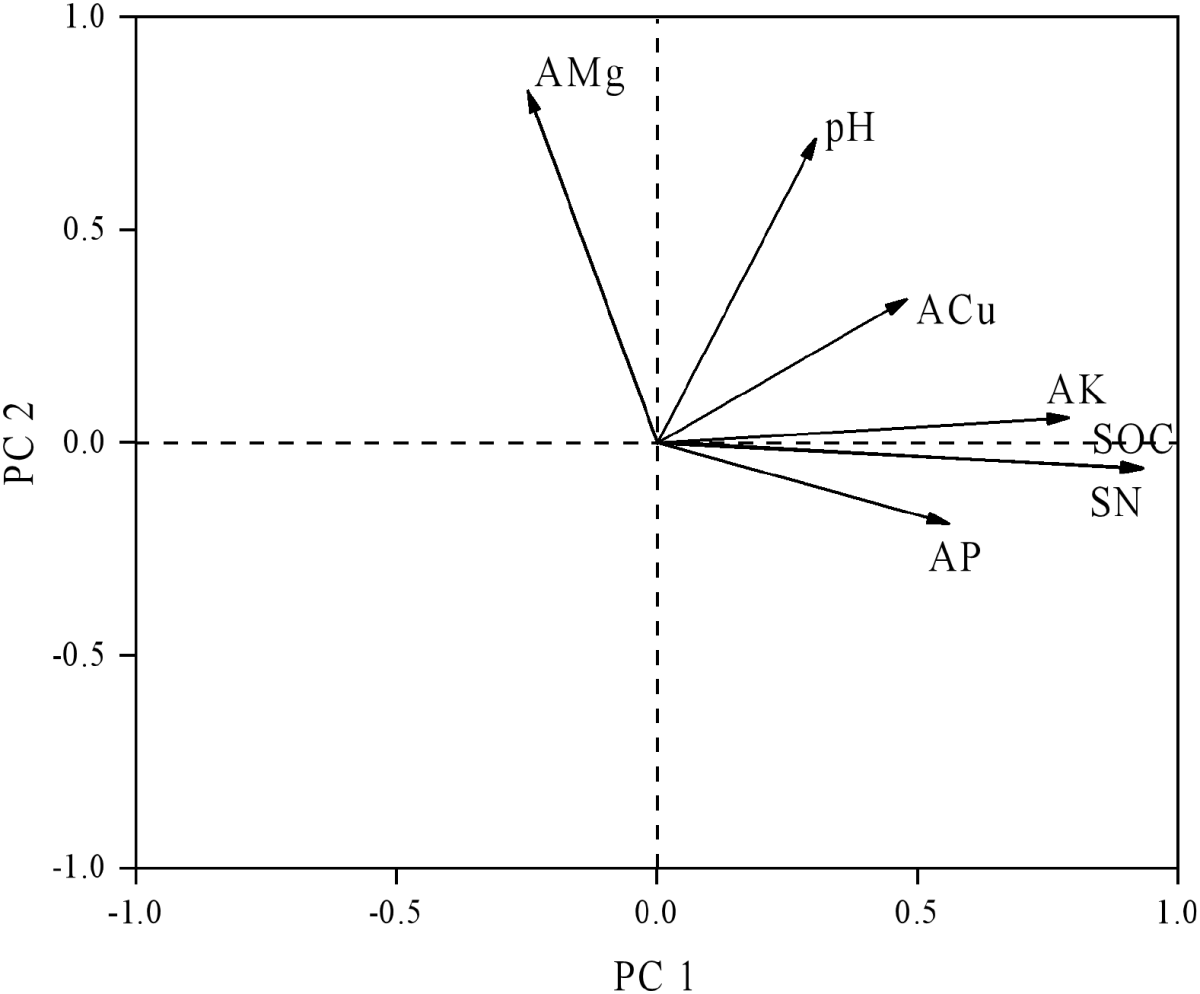
Principal component analysis of soil nutrients. SOC, soil organic carbon; TN, total nitrogen; AMg, available magnesium; ACu, available copper; AK, available potassium; AP, available phosphorus.

**Figure 5:**
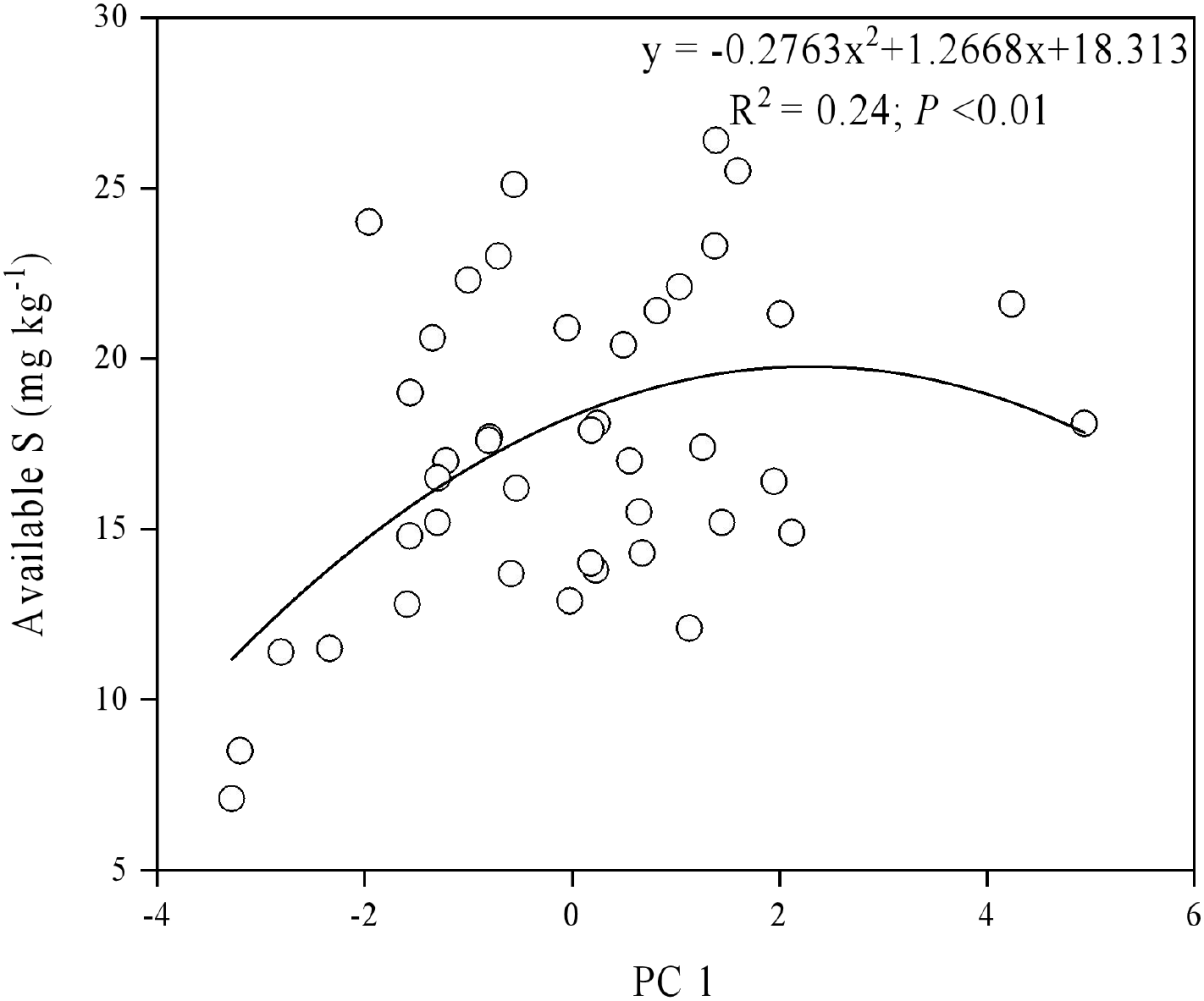
Correlations between principal component (PC1) and available S.

Using a second-order polynomial function, available S was found to be negatively correlated with SOC, SN, and available K but showed no significant difference from soil pH, available P, and available Mg (**Figure 3**). The results of PCA indicated that soil SOC, SN, and available K had a higher degree of interpretation for PC1, while soil available Mg, pH, and available Cu had a higher degree of interpretation for the second principal component (PC2) (**Figure 4**). According to the second-order polynomial function analysis, available S showed a significant correlation with PC1 (**Figures 4**, **5**). In the subsequent pairwise correlation analysis between soil available nutrients, pH, SOC, SN, and PC1, SOC, SN, available K, available P, and available Cu were most relevant to PC1 (**Table 3**).

**Table 3:** Discriminant analysis of soil pH, total content of C and N (g kg^−1^), and soil available nutrients (mg kg^−1^).

Parameter	Correlation coefficient							
	Soil pH	SOC	TN	Available P	Available K	Available Mg	Available S	Available Cu
Canonical 1	0.340*	−0.061	−0.118	0.237	0.214	0.458*	−0.757***	0.056
Canonical 2	0.458*	−0.056	−0.070	−0.217	−0.111	0.805***	0.241	0.178

The largest coefficients are in bold. *P < 0.05; ****P < 0.0001.

## 4 Discussion

### 4.1 Changes in plant growth at different elevations in tea plantations

Plant height, width, and diameter are important indices for evaluating plant growth. Elevation is a significant environmental factor that affects the distribution and growth of species. The impact of elevation on environmental conditions includes variations in temperature, water availability, light, and soil nutrients, affecting resource availability. As elevation changes, plants will show different adaptability in terms of growth and metabolism (Conlisk et al., 2017; Louthan et al., 2015). As elevations increase, temperatures decrease, precipitation and air humidity rise, and water availability generally improves, reducing water stress on plants (Soomro et al., 2024). In this study, tea plant height and width significantly decreased with the elevation increase, while the ground diameter showed a decreasing trend, albeit not significantly. This negative correlation between altitude and plant height and coarseness is consistent with previous findings (Pellissier et al., 2010), indicating that altitude has a strong effect on the growth of tea plantations. There are various hypotheses about the factors limiting tree growth at high altitudes, with lower temperatures being a common explanation. Research suggests that freezing at high altitudes can lead to poorer water conditions, thereby limiting tea growth (Fang et al., 2021).

### 4.2 Distribution of soil available sulfur at different elevations in tea plantations

Compared to other soil elements, available S is more sensitive to changes in the ecological environment. Despite its low content, S plays a vital role in plant growth (Shi et al., 2016). The decrease in S content with increasing altitude is likely due to a reduced soil temperature, which reduces soil microbial activity. Furthermore, the availability and decomposability of substrates releasing S also influence soil available S levels (Xiao et al., 2015). S deposition is a critical component of soil S cycling (Xiao et al., 2015), and changes in deposition levels with elevation gradients may alter available S levels. When comparing the dynamic changes of sulfur in different ecosystems, forest and farmland systems exhibit significant differences. The distribution of sulfur forms in forest soil is significantly affected by acid deposition, with soil acidification intensifying in high sulfur deposition areas. Sulfate leaching is accompanied by loss of base ions, resulting in nutrient deficiency. In contrast, soil acidification in farmland is mainly caused by intensive agricultural activities (such as fertilizer use), and its sulfur dynamics are more regulated by human management measures. Besides, the altitude difference affects the AS content of tea gardens through multiple mechanisms: in high-altitude areas (>600m), low temperatures (decreasing by 0.5 °C for every 100m increase) inhibit microbial activity, resulting in a significant decrease in organic sulfur mineralization rate. However, the enhanced adsorption of iron and aluminum oxides increases the proportion of fixed sulfur; The alternation of dry and wet conditions at mid altitude (100-500m) promotes the sulfur redox cycle; Heavy precipitation (>1200mm/yr) in low altitude areas leads to 60% higher sulfate leaching than in high altitude areas. In addition, microbial activity, ecosystem species composition, and productivity also affect available S distribution (Li et al., 2017).

The content of available P and K increases with elevation due to several factors. Low-altitude areas experience more human interference, leading to significant leaching losses of available P and K. In contrast, the lower temperature reduces the absorption and utilization of available P and K by tea plants. Tea plantations thrive in acidic and slightly acidic yellow and red soils. Soil acidification in tea gardens is affected by several factors, including the secretion of acidic substances by tea plant roots and the use of fertilizers, along with increased acid deposition. In our study, the soil pH of tea gardens ranged between 4.12–4.31, lower than the optimal pH range of 4.5 to 6.5 for tea growth. When soil pH falls below 4.0, tea plant growth and development are inhibited, affecting tea quality and yield. Therefore, addressing soil acidification in tea gardens is crucial.

Discriminant analysis showed significant changes in available S and Mg with altitude (**Figure 2**; **Table 4**), revealing their importance in nutrient management and evaluation in tea plantations at different elevations (Nguyen and Goh, 1992). Amino acids, crucial for tea quality, are influenced by the application of S and Mg, highlighting the importance of magnesium sulphate for tea quality (Ruan et al., 1998).

**Table 4:** Pairwise correlations between principal component (PC1) and soil available nutrients (mg kg^−1^), pH, soil organic carbon (SOC, g kg^−1^), and soil total nitrogen (SN, g kg^−1^).

Variable	PC1	
	R	P
pH	0.305	0.049
SOC	0.933	<0.0001
TN	0.931	<0.0001
Available P	0.559	0.0001
Available K	0.790	<0.0001
Available Mg	−0.249	0.112
Available Cu	0.478	0.001

### 4.3 Relationship between available S and other nutrients in tea plantation soil

The stoichiometry between available S and other elements indicates that other nutrients can influence available S (Shi et al., 2016; Tipping et al., 2016). In our study, available S showed a significant correlation with SOC, SN, and available K (**Figure 3**), supporting the notion that S is an important component of soil organic matter (Biederbeck, 1978). Our findings further validate this view regarding effective nutrients. Soil S exists in organic and inorganic forms, cycling through oxidation, reduction, mineralization, and fixation processes (Wilhelm Scherer, 2009). Available S, which includes adsorbed S, soluble S, and some organic S, is the main source of plant S (Yang et al., 2007) and effectively reflects the soil-plant nutrient status.

Studies on the C: N: S ratios in Australian and global soils have shown that the stable C: N: S ratio in soil organic matter is consistent across different soils worldwide (Kirkby et al., 2011). This suggests a fundamental property of the organic material, with a stable relationship between C, N, and S. However, P is mainly derived from the soil parent material, leading to variability in different studies.

The significant correlation between PC1 and available S based on various soil nutrients, including pH, suggests that available S is closely related to soil environmental conditions and major elements (**Figures 4**, **5**) (Chen et al., 2016; Fan et al., 2017). Available S has a strong sensitivity to the soil environment, making it a good indicator of the effect of soil nutrient changes. Pairwise correlations analysis showed that SOC, SN, and available K were most relevant to PC1 (**Table 4**), indicating SOC strongly contributes to PC1 and potentially alters available S across elevation gradients. However, no significant correlation between pH and PC1 was observed (**Table 4**), indicating environmental conditions of microbial communities in the tea gardens may not substantially change within the narrow elevation range investigated in this study. Future studies should consider a larger altitude gradient to examine pH changes and their contributions to nutrient availability.

Biogeochemical processes better predict the soil nutrient stoichiometry than individual biological or geochemical processes (Luo et al., 2015). In non-arid regions, soil available S content correlated positively with SOC and SN, but this correlation disappeared in the arid areas (Luo et al., 2015), indicating that climate is an important factor affecting soil biogeochemical processes. Future research should explore tea plantations in different climatic regions.

Considering the unevenness of sampling, it may exists difficulty in effectively separating altitude effects from local interference factors (Ma et al., 2022). Besides, the limitation of this study is that it did not systematically quantify the differences in tea tree varieties, changes in microbial community structure, and the interactive effects of long-term climate fluctuations on sulfur cycling under different altitude gradients, resulting in uncertainty in the mechanism explanation.

## 5 Conclusions

The soils of tea plantations at different elevations exhibited distinct separation. Correlation analysis between available S and the first principal component (PC1) indicates that available S is closely related to soil environment and elements, suggesting significant changes in available S in response to elevation gradients. Therefore, available S and Mg should be considered in the nutrient management and evaluation of tea gardens at different elevations. Although narrow-range elevation changes did not significantly alter soil environmental conditions, future research should explore higher altitudes to identify influential soil factors. Additionally, future studies should focus on the role of microbial and other biological factors in influencing the differences in soil available sulfur across different elevations. Understanding the mechanisms through which microorganisms affect S availability can provide deeper insights into the nutrient dynamics of tea plantation soils. Furthermore, examining the relationship between S availability and other elements could reveal how differences in biogeochemical cycling processes might impact climate change. Investigating these interactions will be crucial for developing strategies to manage nutrient availability and enhance the sustainability of tea plantations under varying environmental conditions.

## Data Availability

The original contributions presented in the study are included in the article/supplementary material. Further inquiries can be directed to the corresponding authors.
